# sFlT-1/PlGF ratio as a predictor of preeclampsia in COVID-19 pregnant patients

**DOI:** 10.1186/s12884-024-06263-y

**Published:** 2024-01-31

**Authors:** Kamil Pluta, Marcin Januszewski, Laura Ziuzia-Januszewska, Michał Kudan, Maria Suchocka, Kinga Kuśmierczuk, Tadeusz Issat, Artur J. Jakimiuk

**Affiliations:** 1 Department of Obstetrics and Gynecology, National Medical Institute of the Ministry of Interior and Administration, Warsaw, Poland; 2 Department of Otolaryngology, National Medical Institute of the Ministry of Interior and Administration, Warsaw, Poland; 3 Department of Obstetrics and Gynecology, Institute of Mother and Child, Warsaw, Poland; 4 Center for Reproductive Health, Institute of Mother and Child, Warsaw, Poland

**Keywords:** Preeclampsia, Pregnancy, sFlt-PlGF, COVID-19, Hypertension, Gynecology, Prospective studies

## Abstract

The association between SARS-CoV-2 infection in pregnancy and preeclampsia is widely debated in numerous studies. The aim of our study was to investigate whether an increased sFlt-1/PlGF ratio is a good marker of preeclampsia in pregnant patients with COVID-19 infection. This single centre prospective study was conducted in the Department of Obstetrics and Gynaecology, at the Central Clinical Hospital of the Ministry of the Interior and Administration in Warsaw. The study group consisted of 68 COVID-19 pregnant patients and 57 SARS-CoV-2 negative pregnant controls. Serum sFlt-1/PlGF ratio was assessed. The two groups did not differ in terms of the frequency of IVF, nulliparity, history of hypertension, pre-gestational diabetes and chronic kidney disease. The primary outcome was the diagnosis of preeclampsia. Preeclampsia was diagnosed in 10 patients in both groups. The sFlt-1/PlGF ratio higher than 38, considered highly suggestive of developing preeclampsia, was found in 20 patients in the COVID-19 group and 15 patients in the control group. The odds of developing preeclampsia in patients with sFlt-1/PlGF ratio > 38 was approximately 4-fold higher in COVID-19 group and 11-fold higher in controls. Sflt-1/PlGF ratio does not differ significantly between the SARS-CoV-2-positive and SARS-COV-2-negative pregnant patients. The sFlt-1/PlGF ratio > 38 is associated with higher odds of the diagnosis of preeclampsia in both of these groups, and therefore may serve as its marker regardless of COVID-19 infection status.

## Introduction

Since WHO had announced the pandemic of COVID-19 disease caused by the SARS-19 virus on March 11, 2020 [1], it has resulted in many deaths among pregnant women and has become the main factor adversely affecting the pregnancy trend [2]. Numerous studies have shown that pregnant women with COVID-19 have higher risk of preeclampsia, preterm birth, and fetal death [3, 4]. Preeclampsia is associated with significant maternal and fetal morbidity and mortality affecting 2–7% of all pregnancies. The discovery of circulating angiogenic factors in the pathogenesis of preeclampsia represents an important advance in both diagnosis and prognosis. The soluble anti-angiogenic factor fms-like tyrosine kinase 1 (sFlt-1) and the proangiogenic factor, placental growth factor (PlGF) can be detected and measured in plasma and are usually reported as a ratio [5, 6].

**Fig. 1:**
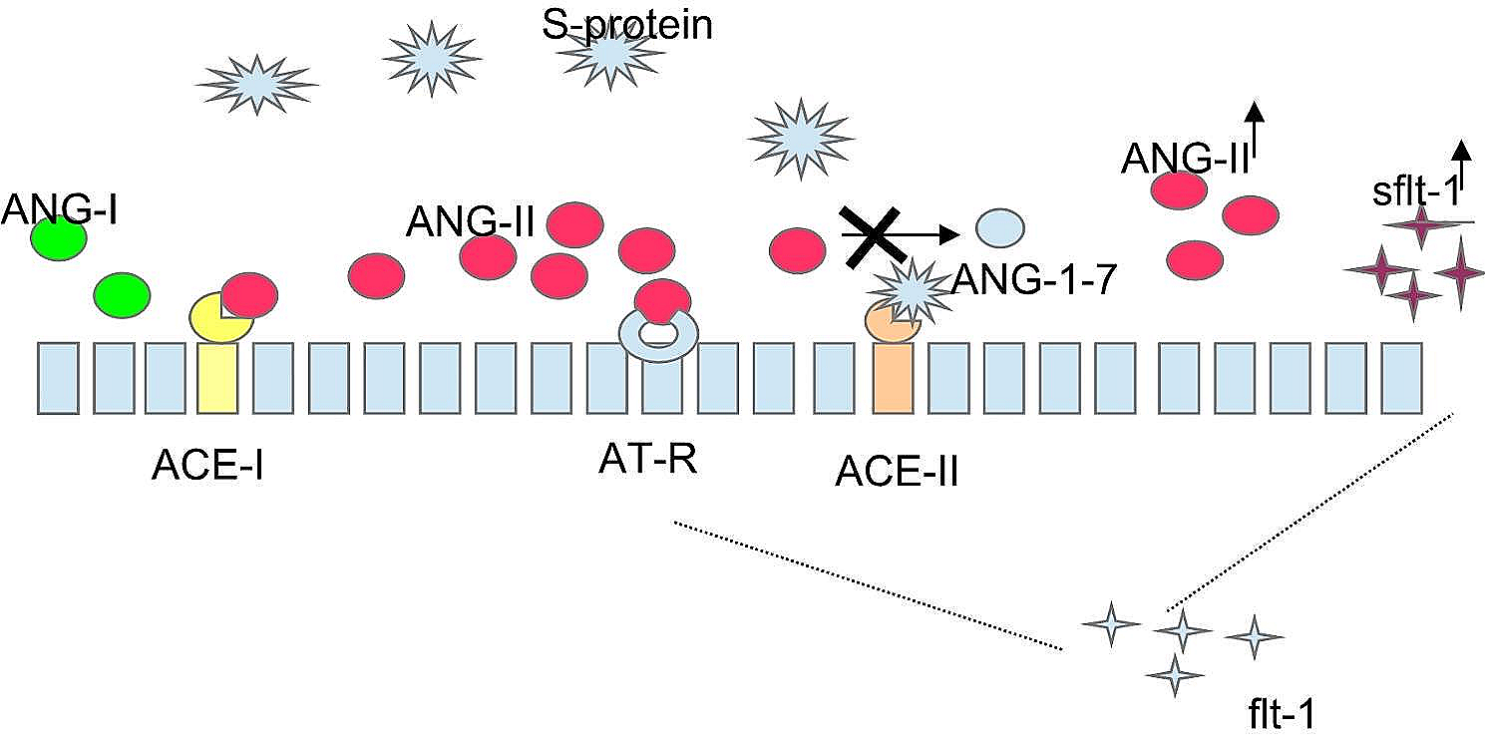
Spike protein effects on RAS

It is known that the Renin-Angiotensin system “RAS” plays an essential role in the pathogenesis of COVID-19 and preeclampsia [7, 8]. In this system, renin cleaves angiotensinogen into Angiotensin I (ANG I), then ANG I is converted into Angiotensin II (ANG II) by the Angiotensin-Converting Enzyme-1 (ACE 1), and ANG II is transformed into ANG (1–7) by Angiotensin-Converting Enzyme-2 (ACE 2) [9]. RAS components are present in the trophoblast and contribute to placental invasion, circulation, and angiogenesis during normal pregnancy [10]. Normal pregnancy is characterized by a relative insensitivity to ANG II, allowing low systemic vascular resistance [11]. In target tissues (alveolar epithelial cells, intestinal epithelial cells, and endothelial cells) spike protein of SARS-CoV-2 binds to and downregulates ACE 2, resulting in increased ANG II levels. Angiotensin II promotes the release of soluble fms-like tyrosine kinase 1 (sFlt-1) by binding to type-1 receptor (Fig. 1) [12]. Preeclampsia represents a model of ANG II mediated endothelial dysfunction. Trophoblasts are resistant to ANG II during normal pregnancy, but remain sensitive in women who later develop preeclampsia. Several studies have shown an imbalance between angiogenic factor (PlGF) and antiangiogenic factor (sFlt-1) [13]. Therefore sFlt-1, a soluble inhibitor of vascular endothelial growth factors (VEGFs), is induced by Ang II Type 1 receptor (AT1) activation by ANG II in response to hypoxia [14]. sFlt-1 is an endothelial decoy receptor that acts as a trap for VEGFs, like placental growth factor (PlGF). Futhermore, sFlt-1 mediates endothelial damage by impairing nitric oxide (NO) production and, more importantly, it sensitizes endothelial cells to ANG II, [15] thus starting a positive loop.

Recent studies have shown strong association between COVID-19 and occurence of preeclampsia. Meta-analysis conducted by Agudelo found that SARS-CoV-2 infection during pregnancy was associated with a significant increase in the odds of preeclampsia (pooled odds ratio, 1.58; 95% confidence interval, 1.39–1.80; *P* < 0.0001; І2 = 0%; 11 studies) [16].

The effects of COVID-19 on pregnancy outcomes were reported by Papageorghiou et al. The study consisted of 2184 pregnant women. COVID-19 was diagnosed in 725 (33.2%) of them. After the adjustment for the sociodemographic factors and conditions associated with both COVID-19 and PE, the risk ratio for PE was significantly higher in COVID-19 group ratio (RR) 1,77, 95% CL 1,25 − 2,52) [3].

COVID-19 is primarily a respiratory infection. It has significant systemic effects including hypertension, renal disease, thrombocytopenia, and liver damage. These signs and symptoms of SARS‐CoV‐2 infection are belived to be caused by vasoconstriction resulting from the dysfunction of the renin‐angiotensin system [17]. In turn, the clinical symptoms of preeclampsia (PE) are mainly a consequence of the endothelial damage caused by oxidative stress in the placenta and antiangiogenic state, leading to hypertension and proteinuria, increased activity of liver enzymes, renal failure or thrombocytopenia, among others. In some of these cases misdiagnosis may have occurred because the clinical symptoms of COVID‐19 and PE overlap. Therefore, differential diagnosis may be difficult in pregnant women with COVID-19 who suffer from hypertension and proteinuria, thrombocytopenia or elevated liver enzymes. With these findings, our aim of this study was to determine whether the sFlt-1/PlGF ratio is independent marker of PE in pregnant women with COVID-19.

## Materials and methods

### Study protocol

This single-centre prospective study was conducted in the Department of Obstetrics and Gynecology, at the Central Clinical Hospital of the Ministry of the Interior and Administration in Warsaw between March 2021 and August 2022.The study group consisted of 68 pregnant patients with COVID-19 infection and 57 pregnant women with a SARS-CoV-2 negative control group. All women were confirmed to have SARS-CoV-2 infection by RT-qPCR testing. The control subjects were pregnant with a nagative SARS-CoV-2 RT-qPCR test who were admitted to the department during the same period. The following data were collected: age, body mass index (BMI), gestational age, parity, smoking, in vitro fertilization, chronic hypertension, chronic renal disease, pregestational diabetes mellitus (PGDM), mean arterial pressure (MAP). The results of the following serum biochemical tests were collected and analyzed: complete blood count, prothrombin time (PT), activated partial thromboplastin time (aPTT), C-reactive protein (CRP), general urine test, protein in the urine, alanine aminotransferase (ALT), aspartate aminotransferase (AST), creatinine, uric acid, lactate dehydrogenase (LDH), platelet count, D-dimer, placental growth factor (PlGF), sFlt-1, and sFlt-1/PlGF ratio. Each fetus was examined by ultrasound. Estimated fetal weight, amniotic fluid, umbilical artery doppler, middle cerebral artery doppler and uterine artery doppler.

were collected.

### Study outcome

The outcome was the diagnosis of preeclampsia (PE). Chronic hypertension (CH), pregnancy inducted hypertension (PIH) and PE were diagnosed according to the guidelines of the Polish Society of Obstetricians and Gynaecologists. Chronic hypertension with the onset prior to conception or before 20 gestational weeks usually persists for over 6 weeks postpartum. PIH is a new onset of hypertension after 20 gestational weeks, not concomitant with proteinuria, biochemical and haematological abnormalities. PIH usually resolves within 6 weeks postpartum. PE is a new onset of HT after 20 gestational weeks plus new onset proteinuria and/or maternal kidney injury, maternal liver injury, neurological symptoms, haemolysis or thrombocytopenia and/or IUGR [18]. Preeclampsia was diagnosed in the presence of hypertension and organ damage, or fetal growth restriction or Doppler abnormalities. Body mass index (BMI) was calculated by dividing the actual body mass by the square of the body height.

### Statistical analysis

Statistical analysis was performed using Statistica software (version 13.3; StatSoft, Poland). A two-sided *p*-value < 0.05 was considered statistically significant. As most of the continuous variables were non-normally distributed, they were reported as median and interquartile range (IQR) and compared with a Mann–Whitney U-test. Categorical variables were presented as the number of patients and percentages and compared with the chi-squared test with Yates correction or Fisher’s exact test as appropriate. Univariate logistic regression analyses were performed to determine the association between sFlt-1 /PLGF ratio > 38 and preeclampsia diagnosis [19].

### Ethical issues

The study protocol was approved by the Bioethics Committee at the Central Clinical Hospital of the Ministry of the Interior and Administration in Warsaw (Decision number.25/2021). All enrolled women gave a written informed consent to participate in the study.

## Results

In both groups, there were no statistically significant differences in the occurrence of risk factors for preeclampsia: BMI, first pregnancy, IVF, smoking, chronic kidney disease, chronic hypertension, pregestational diabetes, except median age, which was higher in control group. The basic characteristics of the study and control groups are presented in Table 1.

**Table 1:** Patients characteristics

Variable	Control group	Study group	p
Number, of patients n(%)	57 (45,6)	68 (54,4)	
Age, years, median (IQR)	37(30,5;40)	33(29;36)	0,014*
COVID-19 severity, n (%)		68 (100)	
1- mild illness		59 (86,76)	
2- moderate illness		8 (11,76)	
3- severe illness		1 (1,47)	
4- critical illness		0 (0)	
Body mass index(kg/m2) median (IQR)	28,57 (25,69;31,005)	29,660 (26,447;33,453)	0,29*
First pregnancy n(%)	32 (56,1)	33 (48,5)	0.504**
IVF, n (%)	10 (17,5)	6 (8,8)	0.236**
Smoking, n (%)	0 (0)	2 (2,9)	0,5***
PGDM, n (%)	1 (1,75)	2 (2,9)	1,0***
Chronic hipertension, n (%)	2 (3,5)	3 (4,4)	1,0***
Chronic renal disease, n (%)	1 (1,75)	0 (0)	0.456***

*Mann–Whitney U-test,**chi-squared test with Yates correction,***Fisher’s exact test

Median age (IQR) in control group was statistically higher − 37(30,5;40) than in study group – 33 (29;36). In both groups, there were no statistically significant differences in the occurrence of risk factors for preeclampsia such as: body mass index, median (IQR) in control group 28,57 (25,69;31,005) and in study group 29,660 (26,447;33,453), *p* = 0,29; smoking, n (%) in control group 0(0), in study group 2 (2,9) *p* = 0,5; IVF, n (%), control group 10 (17,5) in study group 6 (8,8), *p* = 0,236 ; nulliparity n(%), control group 32 (56,1) vs. study group 33 (48,5), *p* = 0,504; PGDM, n (%) 1 (1,75) vs. 2 (2,9), *p* = 1,0; chronic hipertension, n(%) control group 2 (3,5) vs. 3 (4,4), *p* = 1,0; chronic renal disease, n (%) in control group 1 (1,75) and 0 (0) in study group, *p* = 0456. Mild, moderate, severe and critical COVID-19 accounted for *n* = 59 (86,76), *n* = 8 (11,76), *n* = 1 (1,47) and *n* = 0 (0%) cases, respectively. In both groups, there were no statistically significant differences in the occurrence of adverse complications such as diagnosis of preeclampsia, hypertension, HELLP, eclampsia, DIC or increased incidence of caesarean section. Data od adverse outcome is presented in Table 2.

**Table 2:** Pregnancy outcomes

Variable	Control group	Study group	p
Number of patients, n (%)	57 (45,6)	68 (54,4)	
cesarean section, n (%)	18 (31,57)	23 (33,82)	0.707**
Diagnosis of hipertension, n (%)	25 (35,08)	26 (38,23)	0.677**
Clinical symptoms of preeclampsia, n (%)	6 (10,52)	5 (7,35)	0.799**
Diagnosis of preeclampsia, n (%)	10 (17,54)	10 (14,7)	0.852**
HELLP, n (%)	0(0)	1 (1,47)	1***
Eclampsia, n (%)	0(0)	0(0)	
DIC, n (%)	0(0)	0(0)	
sFlt-1/PlGF > 38, n (%)	15 (26,31)	20 (29,41)	0.854**
Odds of developing PE in patients with sFlt-1/PlGF > 38 (OR)	11,38	4.71	0.030**** 0,002****

**chi-squared test with Yates correction, ***Fisher’s exact test, ****Univariate logistic regression analysis

Cesarean section, n (%) was performed in 18 (31,57%) patients in control group and 23 (33,82%) in study group, *p* = 0,707. In control group 25 (35,08) patients met the criteria of hipertension, n(%) in study group 26 (38,23), *p* = 0,677. In both groups there was no case of eclampsia or DIC.

Clinical symptoms of preeclampsia, n (%) were found in 6 (10,25) patients of control group and 5 (7,35) in study group, *p* = 0,799.

Preeclampsia was diagnosed in 10 patients in both groups (14,7% of the patients in COVID-19 group and 17,54% in control group). The sFlt-1/PlGF ratio higher than 38, considered suggestive of developing preeclampsia, was found in 20 patients (29.41%) in the COVID-19 group and 15 patients (26.31%) in the control group. The odds of developing preeclampsia in patients with sFlt-1 /PlGF ratio > 38 was approximately 4-fold higher in COVID-19 group (OR = 4.71; 95% CI 1.16–19.13, *p* = 0.030) and 11-fold higher in controls (OR = 11.38; 95% CI 2.41–53.69, *p* = 0.002).

## Discussion

The reason for this study is that many studies have found an increased incidence of PE among pregnant women diagnosed with COVID-19 and difficulties in differential diagnosis as both diseases can cause systemic endothelial damage, inflammation and multiple organ failure. A correct differential diagnosis is of great importance for the further pregnancy management.

The concept of suspected preeclampsia was first proposed in the original RoPE study [20]. In patients with different pre-existing risk profiles, angiogenic biomarkers predict adverse outcomes, and several clinical trials have established cut-off points for ratios. In the PROGNOSIS (Prediction of Short-Term Outcomes in Pregnant Women Suspected for Preeclampsia) trial, a cutoff value of sFlt-1/PlGF ratio < 38 excluded preeclampsia at 1 week (negative predictive value [NPV] 99.3%) or 4 weeks (NPV 94.3%), while in preeclampsia within 4 weeks coefficient values greater than 38 predominate (positive predictive value [PPV] > 36%) [19].

Endothelial dysfunction caused by SARS-CoV-2 is one of the possible mechanisms for the development of PE in affected women. In recent studies, the virus has been considered as one of the possible etiological factors for the development of PE [21]. According to this hypothesis, SARS-CoV-2 binds to ACE 2 and promotes ANG II, blocking its conversion to ANG 1–7. Because ANG II promotes the release of sFlt-1, the virus is thought to induce an increase sFlt-1 in the blood.

This may lead to a similar occurrence of symptoms such as proteinuria, elevated liver enzymes, inflammatory markers and thrombocytopenia in pregnant women with COVID-19 and in pregnant women diagnosed with preeclampsia [22]. 

Many recent research data published on this subject confirm increased sFlt-1/PlGF ratio in COVID-19 positive pregnant women.

Meta-analysis conducted by Kosińska-Kaczyńska et al. included of 7 studies, revealed that sFlt-1/PlGF ratios between COVID-19 positive vs. negative women were higher, 45.8 ± 50.3 vs. 37.4 ± 22.5, respectively (SMD = 1.76; 95% CI: 0.43 to 3.09; *p* = 0.01) [23].

In another Polish retrospective study conducted by Malicka et al., COVID-19 was associated with higher sFlt-1/PlGF ratio. Authors compared group of 138 SARS-COV 2 positive pregnant and 140 negative controls, and observed significant differences in sFlt-1/PlGF ratio between SARS-CoV-2-positive 24 (3,7–51,5) and negative women 11,2 (2,1–23,4) p-value < 0,01 [24].

Norbega et al. investigated 97 pregnant women. SFlt-1/PlGF ratio was significantly higher in the COVID-19 positive/PE positive group compared to COVID-19 positive/PE negative group (p-value = 0.005), with no increase in cases complicated by SARS [25].

Giardini et al. Identified significant variations in the sFlt-1/PlGF ratio between non—pregnant patients with and without COVID-19.

However, it’s important to note that the study was limited by relatively small number of subjects [26].

In addition, various studies that have investigated sFlt-1/PlGF ratio, as a potential indicators of disease progression of adverse pregnancy outcomes, have yielded conflicting and inconclusive results.

Solvadini et al. conducted a study to investigate the prevalence of hypertensive disorders of pregnancy (HDP) in patients affected by COVID-19. Their research confirmed a significantly higher occurrence of HDP in pregnancies affected by COVID-19 when compared to a control population. It’s worth noting that the sFlt-1/PlGF ratio was found to be higher in HDP patients, whether they had a SARS = CoV-2 infection or not. However, in this study, the sFlt-1/PlGF ratio did not prove to be helpful tool in differentiating the severity of this infection [27]. Futhermore, a study conducted by Malicka et al. revealed that the sFlt-1/PlGF ratio was higher in pregnant women with severe COVID-19 disease (50,8 vs. 16.2; *p* < 0,01). Nevertheless, it did not exhibit significant differences among patients with non-adverse and adverse outcomes, including maternal death, admission to the intensive care unit, multiple organ failure in the mother, preterm delivery, fetal demise, preeclampsia or post- COVID-19 hypertension [27].

Our study has important clinical implications, as we show that sFlt-1/PlGF allow PE to be differentiated from PE-like syndrome present in some of the pregnant women with COVID-19.

## Conclusions

SFlt-1/PlGF ratio does not differ significantly between the SARS-CoV-2-positive and SARS-COV-2-negative pregnant patients. The sFlt-1/PlGF ratio > 38 is associated with higher odds of the diagnosis of preeclampsia in both of these groups, and therefore may serve as its marker regardless of COVID-19 infection status. Pregnant women with COVID-19 could develop a PE‐like syndrome, which might be distinguished from an actual PE by sFlt‐1/PlGF ratio assessment as our study shows. Therefore, healthcare providers should be aware of its existence and monitor pregnancies with suspected PE with caution. PE‐like syndrome might not be an indication for an earlier delivery in itself, as it might not be a placental complication and could resolve spontaneously after recovery from COVID-19. However, more prospective large studies are required to confirm this relationship.

## Data Availability

The datasets generated and/or analyzed during the current study are not publicly available due to our policy but are available from the corresponding author upon reasonable request.
